# The next generation of healthcare ecosystem in the metaverse

**DOI:** 10.1016/j.bj.2023.100679

**Published:** 2023-12-02

**Authors:** Yong Li, Dinesh Visva Gunasekeran, Narrendar RaviChandran, Ting Fang Tan, Jasmine Chiat Ling Ong, Arun James Thirunavukarasu, Bryce W. Polascik, Ranya Habash, Khizer Khaderi, Daniel S.W. Ting

**Affiliations:** a Singapore National Eye Centre, Singapore Eye Research Institute, Singapore, Singapore; b The Ophthalmology & Visual Sciences Academic Clinical Programme, Duke-NUS Medical School, Singapore, Singapore; c Yong Loo Lin School of Medicine, National University of Singapore, Singapore, Singapore; d Department of Pharmacy, Singapore General Hospital, Singapore, Singapore; e School of Clinical Medicine, University of Cambridge, Cambridge, UK; f Wake Forest University School of Medicine, Winston-Salem, North Carolina, USA; g Bascom Palmer Eye Institute, University of Miami, Florida, USA; h Department of Ophthalmology, Stanford University, California, USA

**Keywords:** Metaverse, Healthcare, Ecosystem, Artificial intelligence, Web 3·0

## Abstract

The Metaverse has gained wide attention for being the application interface for the next generation of Internet. The potential of the Metaverse is growing, as Web 3·0 development and adoption continues to advance medicine and healthcare. We define the next generation of interoperable healthcare ecosystem in the Metaverse. We examine the existing literature regarding the Metaverse, explain the technology framework to deliver an immersive experience, along with a technical comparison of legacy and novel Metaverse platforms that are publicly released and in active use. The potential applications of different features of the Metaverse, including avatar-based meetings, immersive simulations, and social interactions are examined with different roles from patients to healthcare providers and healthcare organizations. Present challenges in the development of the Metaverse healthcare ecosystem are discussed, along with potential solutions including capabilities requiring technological innovation, use cases requiring regulatory supervision, and sound governance. This proposed concept and framework of the Metaverse could potentially redefine the traditional healthcare system and enhance digital transformation in healthcare. Similar to AI technology at the beginning of this decade, real-world development and implementation of these capabilities are relatively nascent. Further pragmatic research is needed for the development of an interoperable healthcare ecosystem in the Metaverse.

## Highlights

• The Metaverse combines extended reality, AI, blockchain, Internet of Things, 5G and 6G.
• The applications in healthcare: avatar-based meetings, immersive simulations, social interactions.
• Patients, healthcare providers, and organizations, can achieve these applications.
• Further research is needed for developing the healthcare ecosystem in the Metaverse.

## 1 Introduction

Digital health innovations are transforming medicine, with change accelerated through necessity during the COVID-19 pandemic [1]. Telemedicine helps reduce pressures on physical clinic capacity, and improves access to healthcare services [2]. Virtual care using telemedicine is primarily delivered using mobile phone calls and cloud-based video conferencing software today [3]. However, patient engagement and satisfaction in their interactions with virtual care or autonomous digital applications can vary depending on clinical context [[4], [5], [6]], potentially hinder long-term patient engagement, and impair therapeutic relationships between patients and their providers. The Metaverse offers innovative solutions to address these limitations, especially for patients facing access barriers due to physical disabilities or geographical distance.

Besides healthcare service delivery, the Metaverse holds significant potential for healthcare applications, including enhance collaboration between clinicians and researchers. While existing technology has facilitated remote communication, challenges in team bonding and creative tasks persist, underscoring the need for more immersive platforms. Clearly, the best is yet to be for existing platforms, and there is room for improvement in simulating aspects of communication dependent on gestures and the physical characteristics of objects.

Virtual reality (VR) and augmented reality (AR) create immersive Metaverse environments, with growing interest as VR and AR technology becomes more accessible, with an estimated 50,000 users in October 2021 [7]. Examining the capabilities and ethical considerations of the emerging Metaverse is critical for healthcare innovation [8], enabling improved collaboration, education, and healthcare service delivery [9]. The latest developments in web-based Metaverse platforms facilitate customization, expanding the scope of potential applications across medicine [10], including multidisciplinary team meetings and conferences. Potential barriers to implementation include cost, accessibility, infrastructure, cybersecurity, and ethical considerations - these must be overcome to develop an effective Metaverse for all [11].

In this study, we define the Metaverse, explore its technical framework, discuss how a virtual healthcare ecosystem may be developed in the context of Metaverse, and describe how such a system may look to patients, healthcare providers, and healthcare organizations. We also outline challenges and potential solutions in the development of the Metaverse, emphasizing its transformative potential in telemedicine, healthcare communication, education, training, and clinical research collaboration, contingent on rigorous development, validation, and governance. Understanding and addressing these challenges are pivotal for the future of healthcare innovation.

## 2 Defining the metaverse

**Fig. 1:**
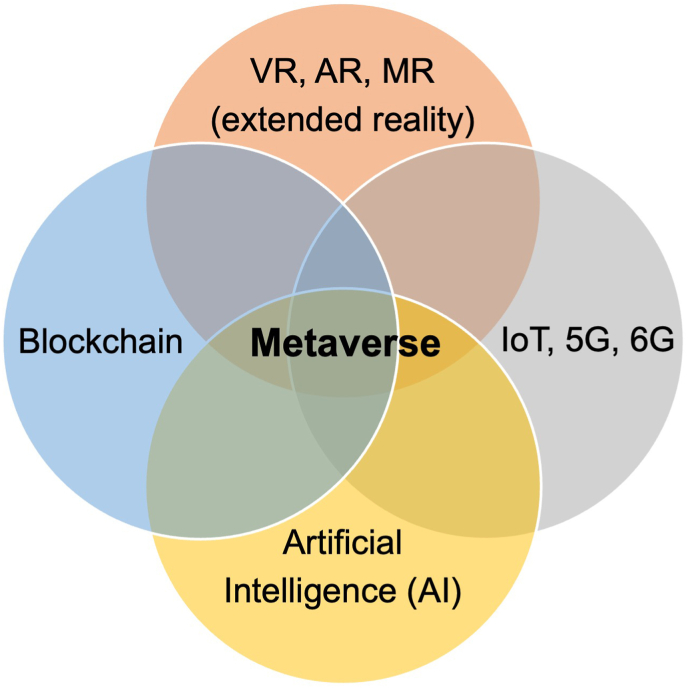
The Metaverse is a technological convergence of extended reality, AI, blockchain, IoT, 5G and 6G technology. VR, virtual reality; AR, augmented reality; MR, mixed reality; IoT, Internet of Things.

As a term, ‘The Metaverse’ was first coined by writer Neal Stephenson in 1992 in the science fiction novel Snow Crash [12], and has seen a surge in interest across sectors in recent years. It pictures a three-dimensional (3D) environment of interconnected virtual spaces, for users to interact freely as avatars and generate content supported by a decentralized economy [Fig. 1] [13]. With ongoing experimentation and incorporation of new technological developments in cryptography, our understanding of the metaverse today has advanced greatly. It has now been established the be the likely application layer for the next generation of the internet, or Web 3·0. The Metaverse can enable immersive digital experiences in extended reality including VR, AR, and mixed reality (MR) through the convergence of advancements in supporting technologies such as AI, blockchain, Internet of things (IoT), 5G and 6G [Fig. 1].

Immersive experiences are now enabled on different interfaces and mobile application platforms, such as mobile smart phones, web desktop, gaming consoles, AR and VR headsets [14]. This is facilitated by the shifting emphasis of leading technology giants like Google, Apple, and Meta (previously known as Facebook) to drive new technological innovations, such as the Oculus Quest 2 VR headset, Vision Pro, and incorporating finger dexterity and haptic feedback tracking by Meta's Reality Labs Research teams [15]. These experiences have opened up a multitude of possibilities to redefine traditional systems across sectors, from sales and retail [16], music and art [17], social networking, journalism [18], to healthcare [9].

Use of digital avatars and digital twins, as physical embodiment of the individual self and physical environments, breaks the boundaries of current 2D platforms to further enhance the sense of self. They also opens opportunities for modelling and prediction of physical processes using its digital counterparts [19]. Another key feature of blockchain-enabled Metaverse platforms is the use of non-fungible tokens (NFTs) [20], that provide a digital warranty of authenticity to allow trustless exchange and secure storage of content in a decentralized manner [9,21]. The foundation for connectivity is the Internet. The rapidly innovations in Internet technology including the 5G and 6G. IoT can also be leveraged in the Metaverse, mapping real-time IoT data from real life into the virtual world. The IoT can also supplement the experience interface of the users into the virtual world created [Fig. 1)] [22,23].

## 3 Technology background

Metaverse applications are user-consumable interactive multimedia experiences that combine immersive 3D renders, distributed real-world data, personalized digital assets, and metadata that bridge the digital and physical worlds.

### 3.1 Immersive technologies

Immersive technologies, including VR, AR, and MR, enable users to perceive and interact with these applications. The level of immersion offered by these technologies depends on the extent to which the human senses are engaged and their hardware capabilities [24]. Typical audio-visual experience can allow users to perceive the digital world visually through volumetric content and aurally through spatial audio in three dimensions. For example, VR can be used for medical training simulations, allowing surgeons to practice procedures in a realistic virtual environment before performing on patients. AR games like Pokémon Go overlay digital creatures onto the real world, creating an interactive gaming experience.

### 3.2 Multisensory immersion

**Fig. 2:**
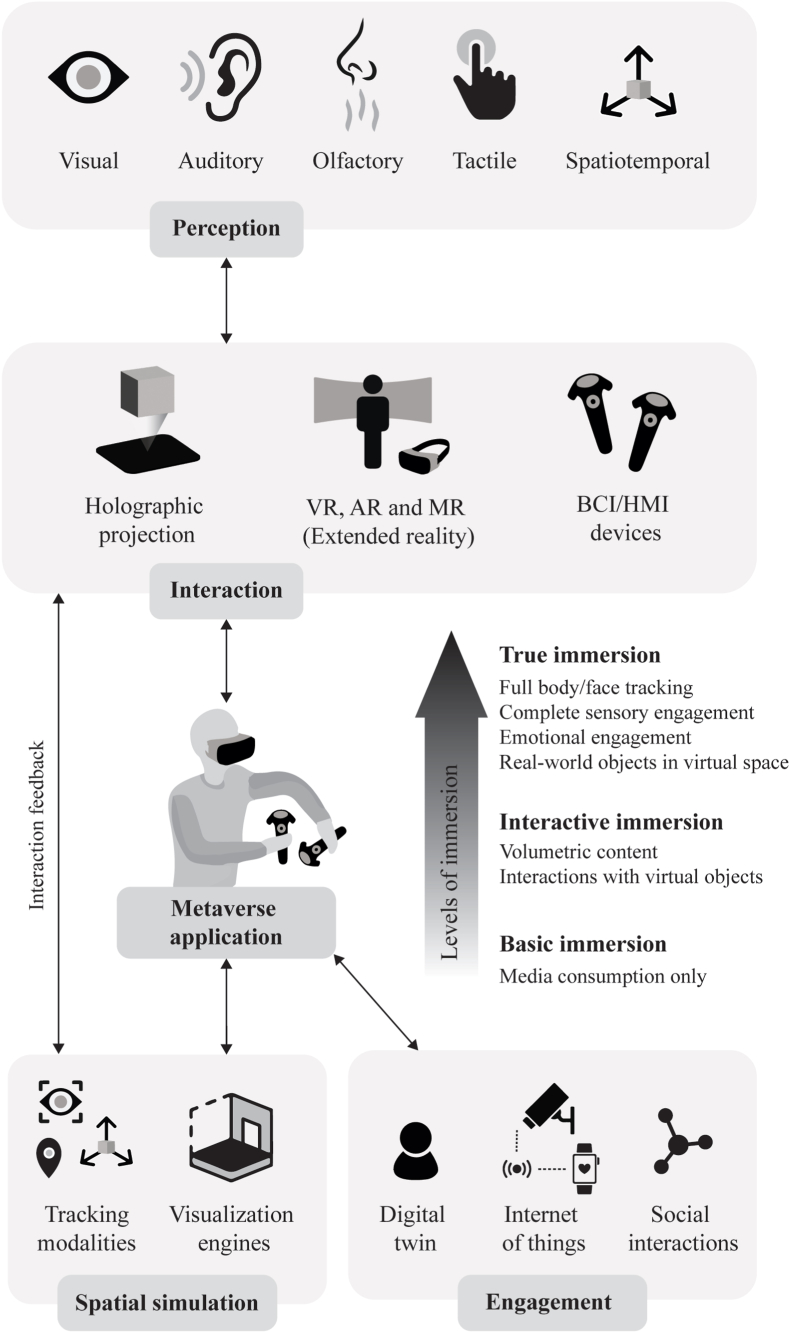
Underlying framework to deliver an immersive Metaverse experience. VR, virtual reality; AR, augmented reality; MR, mixed reality; BCI, brain computer interface; HMI, human machine interface.

The inclusion of haptic feedback and spatial awareness can further augment these experiences. Spatial awareness changes the field of vision and stereo sound perceived by the user based on their position in physical space. At the same time, haptic feedback simulates the sensation of touch while interacting with virtual objects. In addition, digital scent technology can stimulate olfactory perception, allowing users to experience different odors to provide a more immersive, real-world experience. Furthermore, eye-tracking includes eye-facing cameras that collect user data on gazing, facial expressions, and pupil dilations from the real world. They also render realistic avatar reconstructions (digital twin) in the digital world by accurately reflecting their expressions and eye movements. Ultimately, these technologies aim towards a fully immersive multisensory experience to engage users at cognitive and affective levels. Figure 2 illustrates the underlying framework that delivers an immersive multisensory experience.

### 3.3 Near-eye displays (NEDs)

While hardware technologies allow users to interact and immerse themselves, software technologies enable rendering virtual environments and processing user data for a more dynamic experience. Near-eye displays (NEDs) are head-mounted display technologies for AR and VR that have become ubiquitous for metaverse interactions. The display hardware is gaining usability and wider adoption for their performance improvements with the field of view, brightness, spatial and angular resolution. They are also becoming more ergonomic, providing for smaller form factors and mitigating vergence-accommodation conflict [25,26]. NED headsets also integrate several sensors like inertial measurement units (IMUs), Global Positioning System (GPS), light detection and ranging (LIDAR), and head-mounted cameras to facilitate spatial awareness. By utilizing these sensors, they also trigger haptic feedback. Thus, the integration of sensor technology into NED headsets makes interactions via joysticks and pointing devices obsolete and augments visual immersion to the next level [24]. For example, smart glasses like Google Glass overlay digital information onto the user's field of view; devices like the Oculus Rift provide immersive VR experiences with high-quality visuals and tracking capabilities.

### 3.4 Brain-computer interfaces (BCI)

In addition to VR, AR, and MR technologies, holographic displays and brain-computer interfaces (BCI) are also gaining popularity. Holographic projection enables users to see the virtual image with the naked eye at different viewing angles without any wearable device [27]. On the other hand, BCI would decode brain signals via electroencephalography in order to send user commands as a means of interaction in the Metaverse [28]. BCI can potentially transduce user commands and stimulate sensory perception by activating corresponding areas of the brain [29]. Thus, BCI may deliver the most natural form of interaction among these hardware technologies in future. For instance, BCI can assist individuals with paralysis by enabling them to control computers or prosthetic devices through brain signals. It can also offer more natural and immersive control in VR games by interpreting user's thoughts and intentions.

### 3.5 Dara processing and networks

The Metaverse unlocks the true potential of immersiveness by processing a large amount of data at high speeds for content generation. For a comfortable and immersive experience, real-time 3D rendering must be done at 60–90 FPS with very low latency [30]. Cloud computing is deployed to run visualization engines (software applications) that process high-resolution graphics for rendering the virtual world. Whereas real-time processing, such as data from AR/VR devices, gaming commands, haptic feedback, etc., are offloaded to edge devices at the user end. 5G/6G networks are rising as they enhance mobile broadband for personal devices and large-scale IoT infrastructure with high data rates, low latency, and reliability [31].

## 4 Metaverse platforms

Most internet activity currently operates on ‘Web 2·0’, whereby large technology companies develop and provide users access to centralized digital platforms. These platforms have often hosted marketplaces for products and services such as social networks and multimedia streaming [Table 1]. These platforms provide avenues for individuals and businesses to create and share user generated content (UGC). Benefits to users include access to these platforms at little to no cost [32,33]. Many users have made careers out of monetizing UGC shared on these platforms through reviews and podcasts, while others have incorporated use of digital platforms within their core business models and lifestyles [34]. The early users and adopters of these platforms are central to the platforms' popularity and success, which often hinges on the quantity, quality, and variety of UGC contributed within them [35].

**Table 1:** List of web 2.0 legacy and web 3.0 novel metaverse platforms.

Name	Web	Terminal	Dimension	Technical Capabilities	Site Visits	Average Duration
Gathertown	2.0	Mobile, desktop (web-based)	2D	Virtualize physical events; spaces integration between customizable physical avatar-fluid video chat	2.9 M	0:04:23
Engage VR	2.0	Mobile; desktop, VR	2D	Simulate physical interactions, host multi-user events, collaboration, training, education	16.4K	0:03:03
Queppelin	2.0	Desktop, VR	3D	Conduct seminars, meetings, business events in a virtual environment	<5k	NA
serl.io	2.0	Mobile, desktop, MR	3D	Create MR content, deploy in collaborative sessions to engage participants in real-time 3D	<5k	NA
Decentraland	3.0	Desktop, VR	3D	Land NFT for Social Metaverse built on Ethereum chain.	599K	0:07:48
Sandbox	3.0	Desktop (web-based)	3D	Land NFT for Games and Entertainment built on Ethereum and Matic chains.	4.7 M	0:09:35
The Otherside	3.0	Mobile, desktop (web-based)	3D	Land NFT for Games and Social metaverse built on Ethereum chain.	66.3K	0:03:09
NFTworlds	3.0	Desktop (web-based) using Minecraft	3D	Land NFT for Games built on Ethereum chain.	47.7K	0:01:37
Worldwide webb	3.0	Mobile, desktop (web-based)	2D	Land NFT for Games and Social metaverse built on Ethereum chain.	25.7K	0:03:18
Metapolis	3.0	Desktop, VR	3D	Metaverse-as-a-Service (MaaS) built on Ziliqa and for Multi-chain compatibility.	<5K	NA
Somnium space	3.0	Desktop, VR	3D	Land NFT for Games built on Ethereum and Solana chains.	70.5K	0:01:54
Chillchat	3.0	Desktop (web-based)	2D	Origin NFT for Metaverse-as-a-Service (MaaS) built on Solana chain.	7.5K	0:00:11
Portals	3.0	Desktop (web-based)	3D	Land NFT of Social metaverse for sports fans built on Solana chain.	41.3K	0:01:25

Abbreviations: VR: virtual reality; MR: mixed reality; K: thousand; M: million.

However, the low cost of adoption for users comes with several trade-offs, as these digital platforms are reliant on exploiting their users to attain financial sustainability. Firstly, individual privacy is a common trade-off whereby personal data and behavioral patterns of users are collected and monetized by these platforms through sales of data to third parties for the marketing of goods and services [36]. Secondly, new fee structures and paywalls can be added by providers without any notice given to users. These are often predatory and designed to penalize the users with inelastic demand, i.e., users that are most reliant on the platform or most likely to experience disruptions should they lose access to them. Thirdly, these Web 2·0 platforms are operationalized in a permissioned manner. For instance, providers controlling these digital platforms may make unilateral decisions to revoke permissions or access to the platforms. At times, this can be at the detriment of groups of users that may be regular contributors of UGC that enabled these platforms to gain popularity in the first place, even if they might be reliant on the platforms to make a living.

In contrast, ‘Web 3·0’ is centered on the empowerment of individual users through technology. This is enabled by the applications of blockchain, including smart contracts and NFTs. Blockchain technology is increasingly used in healthcare to enhance cybersecurity. It secures patient records by creating an immutable ledger for medical data. This ensures data integrity and reduces the risk of data breaches. Data interoperability is improved as blockchain provides a standardized format for sharing information among healthcare providers. Patients can manage data access through smart contracts, enhancing consent management.

The concept of the Metaverse and Web 3.0 are closely related. They share common goals of decentralization, semantic data, interoperability, trust, and security. The Metaverse can be seen as a prominent use case and a manifestation of the principles and technologies that Web 3.0 seeks to advance, creating a more immersive and interconnected digital experience for users. These two concepts are likely to shape the future of the internet and digital interactions significantly. Examples were collated in [Table 1] for popular applications based on market capitalization or applications with liquid cryptocurrencies [37], as well as transactional volume and unique niche applications based on listings in leading NFT marketplaces [38,39]. In Web 3·0 metaverse applications, users may retain ownership of the UGC that they create, set usage rights for others to repurpose their content, and monetize their content independently. This is facilitated and governed at scale through smart contracts which manage the terms of sale for assets such as UGC. Innovations include programmable royalties collected as a percentage of each sale, at the point of sale. The composability of blockchains also reduces the friction for individuals to switch digital platform providers, given that they own their UGC and can implement it into other platforms. This is quite unlike Web 2·0, where UGC ownership is retained by the technology companies that host UGC on digital platforms, which introduces significant friction for producers and consumers of UGC who wish to switch digital platform providers.

Royalties can be accrued to a treasury of the NFT collection, which can enable owners to vote democratically on the funding of new initiatives and development of new commercial directions within affiliated platforms. This is enabled by the composable and transparent nature of public blockchains, as snapshots of on-chain ownership can be saved at key milestones. This ensures that active stakeholders, who have partial ownership and aligned incentives with the success of the NFT collection, are enfranchised with the ability to participate in the governance and operationalization of these digital platforms.

## 5 Developing an interoperable healthcare ecosystem

**Fig. 3:**
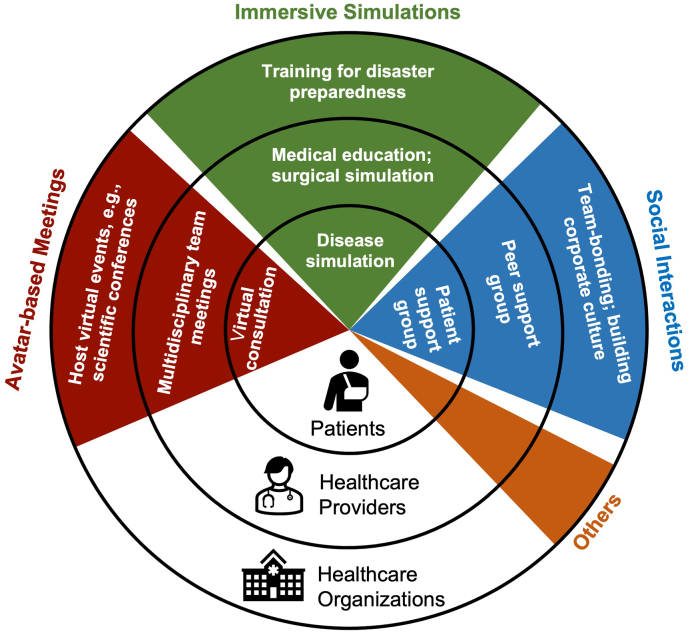
The interoperable healthcare ecosystem in the Metaverse.

Healthcare ecosystems involve a wide range of actors, including patients, physicians, allied health practitioners, state departments, academic institutions, non-governmental organizations (NGOs), and corporations. They incorporate knowledge flows originating from or co-produced by all of these actors with the aim of pursuing a collective goal [40]. For conciseness, discussion focuses here on the three primary actors in the healthcare ecosystem: patients, healthcare providers (physicians, nurses, and other allied health practitioners), and healthcare organizations. The different dimensions of features in the Metaverse, including avatar-based meetings, immersive simulations, social interactions, and others can be applied to all of these actors, creating an interoperable healthcare ecosystem [Fig. 3].

### 5.1 Avatar-based meetings

**Fig. 4:**
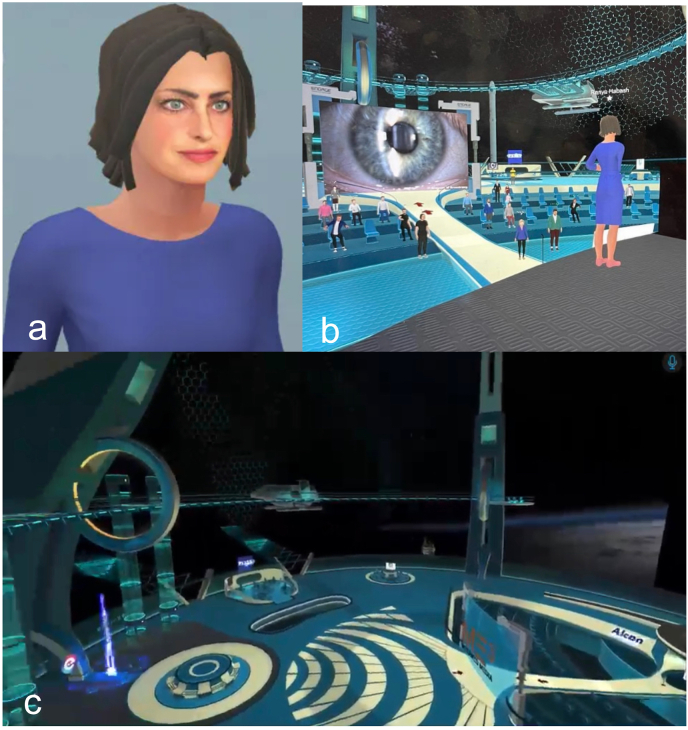
Hosting ophthalmic conferences by Digital Ophthalmic Society in the Metaverse. (**A**) The digital avatar of one of the attendees; (**B**) the experience of the attendee on the conference; (**C**) the virtual setting of the conference.

One of the important features in the Metaverse is the capability to host avatar-based meetings. As the Metaverse integrates all kinds of apps and different dimensions of virtual worlds, the avatar can act as the single point of entry and unique identity, where individuals explore, interact, and engage [Fig. 4a]. With the digital avatars, people can interact with each other and the virtual environment to a much greater extent, compared with conventional online video meetings.

Patients are able to attend virtual consultations in the Metaverse. Traditional online consultations, which are limited to voice or video talk, are often inefficient for real-time communication between patients and medical staff. Compared with video or teleconsultation, deeper interaction and communication with clinicians could be achieved through avatars that mirror their behaviors. VR and AR based virtual diagnostic and therapeutic tools are becoming more tangible through avatars [41,42], creating the realism and “personal-touch” of remote consultations, reducing the requirement for face-to-face interaction. In addition, informed consent for surgery and clinical research includes elements of voluntarism, capacity, disclosure, understanding, and decision [43], which requires the interaction of psychological and intellectual characteristics of an individual. Avatar-based communication with clinicians could enhance patients’ understanding regarding various components of the informed consent process. Lastly, patient outcomes can be improved through VR and AR based digital health tools which offer more successful diagnostic and therapeutic options [44,45].

For healthcare providers, the Metaverse could enhance multidisciplinary team meetings (MDTs). MDTs are patient case discussions involving groups of specialists with diverse expertise relevant to the clinical management of a given patient. These are a core element of collaborative work in hospitals, particularly in caring for clinically complicated patients [46]. The extent of organization and the type of communication in MDTs have a direct effect on the quality of patient care provided [46]. However, traditional face-to-face MDTs are costly in both time and money, with virtual MDTs exhibiting promise to ameliorate these costs [47]. Incorporating participation as avatars, MDTs in the Metaverse can overcome the barriers of traditional online MDTs, including difficulty in building a sense of identity, or misunderstandings that can jeopardize team cohesion. Another advantage of MDTs in the Metaverse is facilitation of the human-computer MDT, which aimed for consultation based on human-computer communication and interaction [48]. This aims to provide comprehensive diagnosis and treatment plans that combine clinicians’ expertise, computerized data, and AI-derived knowledge and guidance.

For healthcare organizations, including hospitals, clinics, institutions, and international medical societies, avatar-based virtual events such as scientific conferences can be hosted in the Metaverse. The advantages of avatar-based virtual events include creating the sense of co-presence which allows for shared experiences in the virtual environments and building deeper connection and interactivity between the avatars. The 3D spatial audio capability even allows avatars to hold intimate conversations based on proximity, just as in live meetings. Recently the Digital Ophthalmic Society, hosted by MetaMed Media, held the first Metaverse ophthalmic meeting, serving as a proof of concept for hosting scientific conferences in the Metaverse [Fig. 4]. These experiences provide numerous benefits over in-person conferences, such as the lack of physical constraints such as venue capacity, and reduced barriers to participation given that expenses for travel and accommodation would not be required.

### 5.2 Immersive simulations

The Metaverse may host more immersive simulation experiences than currently possible, through extended reality incorporating developing technologies; augmenting electronic, digital environments where data are represented and projected [49]. In these extended reality environments, humans interact and observe partially or fully synthetic digital environments constructed by technology.

**Fig. 5:**
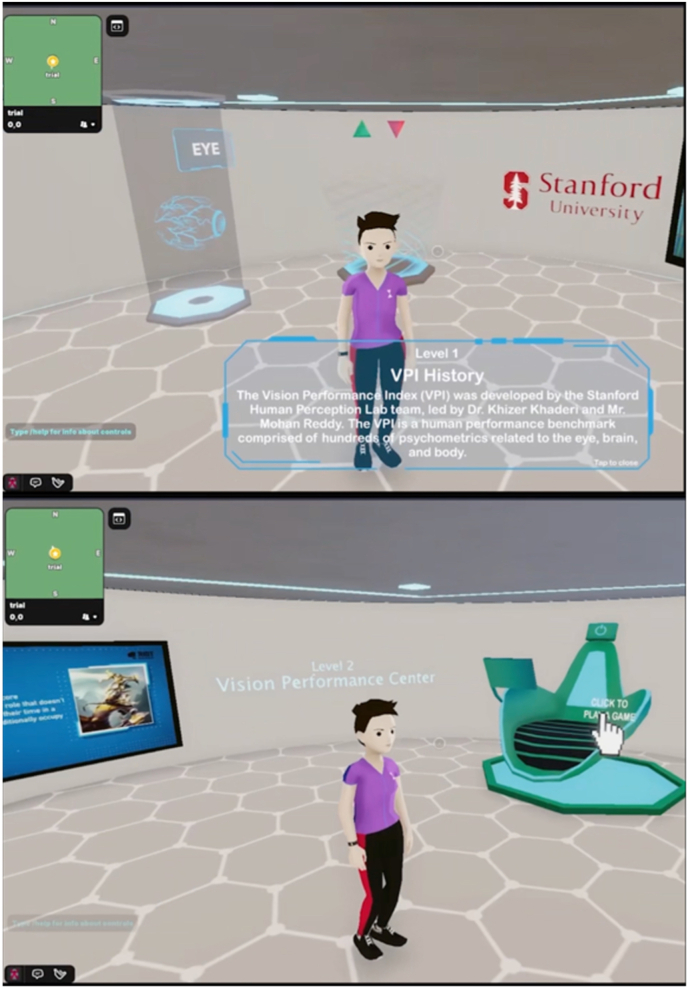
A patient visiting a virtual clinic in the Metaverse.

For patient-centered communication, clinicians need to elicit and understand patient perspectives within their unique psychosocial and cultural context, and finally reach a shared understanding of patient problems [50]. Chronic disease self-management and preventive health programs focus on promoting informed lifestyle choices, risk factor modification, and active patient self-management of chronic diseases [51], which relies heavily on better information and communication practices. The immersive simulation platform in the Metaverse can be applied for disease simulation and patient education. Significant clinical benefits have been identified from self-management or lifestyle interventions across conditions including coronary heart disease, chronic kidney disease, diabetes, and rheumatoid arthritis [51]. In addition, it could also possible to perform clinical examinations and treatment in the Metaverse, such as visual acuity [52], visual function assessment [53,54], psychotherapy [55,56], and rehabilitation therapy [44,45,57]. An example of performing clinical examination in the Metaverse can be found at the Stanford Vision Performance Center at the Byers Eye Institute, where patients can visit a clinic in the Metaverse, and can play videogames enabled to assess visual, cognitive, and motor function via the Vision Performance Index [Fig. 5].

**Fig. 6:**
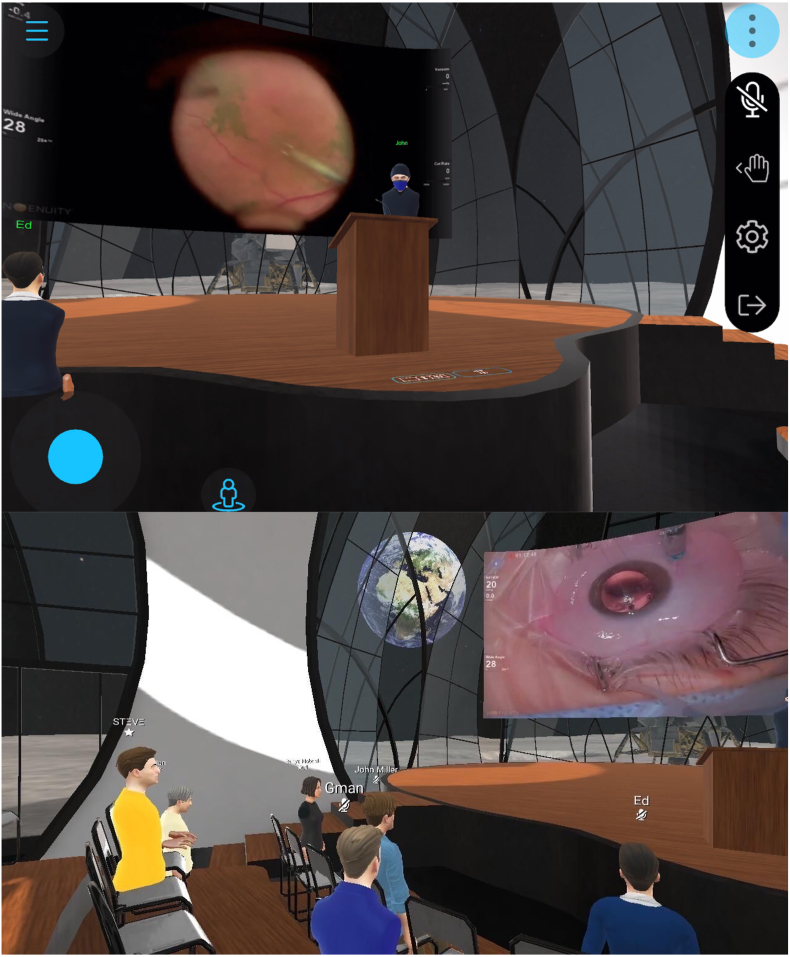
3D ophthalmic surgical rounds host by MetaMed Media.

For healthcare providers, the Metaverse can be applied for medical education, including clinical simulation training, surgical simulation training, and code blue training. A recent prospective study demonstrated that the Metaverse can be an effective system for facilitating and enabling interactions amongst international colleagues for scientific instructions and mentorship [58]. For example, MetaMed Media hosts regular 3D surgical rounds with case discussions between ophthalmology key opinion leaders from across the world and trainees from several academic institutions [Fig. 6]. Furthermore, 3D volumetric capture allows attendees to revisit the event in immersive fashion, similar to the Halliday Journals archive in Ready Player One [59]. With the development of AR and VR devices, the immersive experience is more effective in areas like surgical training that require advanced hand skills and interaction [60]. There are a growing number of studies in the literature that support the efficacy of using VR for surgical training initiatives. For example, a 38 % reduction in cataract surgery complication rates was associated with the use of VR training in a group of first- and second-year surgical trainees [61]. In orthopaedic surgery, it is identified that the use of VR is an effective method of improving technical skill acquisition among trainees learning arthroscopic procedures [62]. Promising findings pertaining to the viability of using extended reality technology for training initiatives have similarly been expressed in plastic surgery [63], cardiothoracic surgery [64], and neurosurgery [65]. Besides medical education, the Metaverse also provides a virtual environment for remote surgeons to conduct actual surgical procedures, such as through surgical robots [66]. This may extend surgical expertise geographically, as already occurring at scale in radiology, allowing high-precision operations to be scheduled around the clock, without reducing accuracy and flexibility [60].

For healthcare organizations, the Metaverse is a promising training platform for disaster preparedness. The preparedness of a hospital for disaster response and mass-casualty incident includes activities, programs and systems developed and implemented before the event [67]. A well-established preparedness program is essential for the effective response to emergencies of healthcare systems. The virtual reality simulation system, for example, has shown to be a viable, cost-effective approach for professional training in in-hospital disaster preparedness [68]. For example, there are emerging virtual simulation methods and platforms designed for pandemic preparedness and response during COVID-19 pandemic [[69], [70], [71]], which could be potentially further upgraded and incorporated into the Metaverse. The reproducibility, repeatability, and real-time training features of the Metaverse platform, in addition to its low cost of implementation, suggest that it could serve as a promising adjunct to conventional approaches to training [68].

### 5.3 Social interactions

Currently, the majority applications of the Metaverse are focused on social and gaming industries. Therefore, enhanced social interactions within the healthcare ecosystem are perhaps the most accessible function of the Metaverse. Besides interactions between the patients and healthcare providers discussed above, the Metaverse also provides virtual platforms for patients' and medical professionals’ communities. Patients may even find them useful to form virtual patient support groups. Studies show that patients benefit from engagement in groups offering emotional support, confidence, strength, and hope; leading to measurable improvements in quality of life [72]. The Metaverse provides the technical infrastructure to create virtual spaces where patients with similar medical conditions or challenges can gather. These platforms may include VR or AR environments, web-based applications, or even mobile apps with augmented reality features. Ensuring the privacy and security of patient data within these virtual support groups is paramount. Utilizing blockchain or decentralized identity systems can help manage user identities and control access to sensitive information. Technical solutions must support real-time interactions, such as voice and video chat, and even gestures within virtual environments. This requires robust network infrastructure and low-latency communication protocols. For healthcare providers, virtual peer support groups could similarly provide accessible emotional support for them by combatting stigma and by creating a supportive forum for healthcare professionals to communicate with each other [73]. Healthcare professionals need secure channels to communicate with each other. Implementing end-to-end encryption and secure messaging protocols can protect sensitive discussions within virtual peer support groups. To ensure that participants in healthcare provider communities are genuine professionals, technologies like digital badges or certificates issued on blockchain can verify their qualifications and credentials. Healthcare organizations may use the Metaverse to host team-bonding activities and build a positive company culture. The Metaverse can facilitate virtual team-building events, workshops, and conferences. These events may involve virtual reality environments, live streaming, and interactive elements like polls and Q&A sessions. Technical aspects include avatar customization to represent users in the Metaverse accurately. Healthcare organizations may vary in size, and technical solutions need to scale to accommodate large numbers of participants in virtual events and activities. Cloud-based infrastructure and content delivery networks (CDNs) can help ensure scalability.

### 5.4 Others

**Fig. 7:**
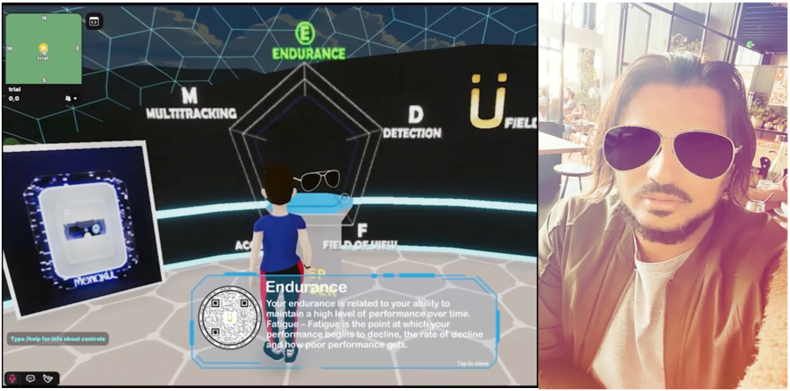
Virtual optical shop in the Metaverse. It can activate an augmented reality try-on and purchase via a QR code.

The Metaverse also includes the retail sector of the healthcare ecosystem. Given early consumer adoption has been focused on social, leveraging mobile devices capabilities via cameras and AR enabled platforms including Instagram and Snap, allows for bidirectional experiences in the Metaverse. An example of such experience is visiting a virtual clinic in the Metaverse, such as the Stanford Vision Performance Center at the Byers Eye Institute [Fig. 7]. Patients are able to visit a virtual clinic where they are provided services, including a virtual optical shop, where one can move from a virtual experience to an augmented experience as illustrated below. The virtual try-on via an AR experience in Instagram allows an individual to explore the Metaverse, interact with experience which can lead to a real-world transaction, including buying a pair of glasses in both the digital world and physical world.

## 6 Challenges and future directions of the metaverse

There remain several potential barriers to widespread implementation of the Metaverse. These include cybersecurity risks, regions with limited access to internet connectivity, lack of technology literacy, and other physical issues that may impair accessibility, such as among visually impaired individuals.

Firstly, the Metaverse carries inherent cybersecurity risks of hacking and exposure of patient privacy data. However, advancement of healthcare organizational cybersecurity protocols has provided potential measures to reduce these risks. For example, the increasing application of blockchain technology could provide “protect and permit” for secure sharing of patients’ data [74]. There have been several reports of healthcare applications of blockchain, with health record management and supporting security within mobile and hardware solutions [75].

Secondly, the increasing bandwidth requirement of immersive experiences in the 3D environment may present challenges for applying these technologies in less technologically developed areas [76]. Sparse internet connectivity has challenged recent efforts to implement AI in healthcare, whereas latency has also been reported as a limitation to implementation in real-world clinical settings [77,78]. Nevertheless, there are emerging solutions to improve connectivity in the field of 5G telecommunications, laying the foundation for a pragmatic avenue to assist application in less-developed areas [79]. These advancements can support the implementation of immersive healthcare solutions and overcome connectivity limitations.

Thirdly, another important barrier to the development of the Metaverse is the learning curve associated with new technology, leading to difficulties in interpreting the user interface, which could lead to clinically significant misunderstandings [[80], [81], [82]]. User-centered design and training programs can ease the learning curve, making immersive technology more accessible and understandable for healthcare professionals. In addition, there may also be issues associated with privacy and corporate overreach relating to commercialization of personal data, as on mainstream Web 2·0 platforms. These are potential barriers to application that may be addressed with focused research aiming to identify stakeholders’ capabilities to apply these technologies and their attitudes towards these solutions [83]. Early research in the field has explored the implementation of immersive technology including AR and VR in healthcare, as most investigated patients show interest in these technological solutions.

Finally, despite the great potential for implementation of immersive technology in healthcare [84], the usage of these technologies for patients with visual or hearing impairment may still remain challenging. For instance, patients with severe visual impairment may not be able to appreciate the full content in a 3D or VR simulation situation [85]. Therefore, advancement of new technology that can customize simulations for patients with special visual or audio needs is warranted to facilitate comprehensive development of the Metaverse.

## 7 Conclusions

The Metaverse, as the anticipated application interface for the next generation of the Internet (Web 3.0), is poised to revolutionize healthcare. In this context, we have defined a vision of the next-generation interoperable healthcare ecosystem within the Metaverse. The Metaverse offers a wealth of possibilities for healthcare, from avatar-based meetings facilitating remote consultations to immersive simulations aiding medical training and social interactions fostering patient engagement. To unlock the full potential of the Metaverse in healthcare, we must address several critical factors. These include the need for continuous technological innovation to refine and expand Metaverse capabilities, the requirement for robust regulatory supervision to ensure data security and ethical use cases, and the implementation of sound governance structures to guide this evolving digital landscape. The concept and framework we propose for the Metaverse have the potential to redefine traditional healthcare systems and accelerate digital transformation in the field. While the Metaverse is currently nascent, its potential is huge; comparable to AI technology at the beginning of the 2010s. Further research into technology, security, and applications is necessary for the real-world development of an interoperable healthcare ecosystem in the Metaverse.

## Author contributions

All authors conceptualised the manuscript, researched its contents, wrote the manuscript, edited all revisions, and approved the final version. Authors YL and DVG are co-first authors that contributed equally to the manuscript.

## Funding

This work was supported by the following funding: 10.13039/501100001349National Medical Research Council, Singapore: MOH-000655-00 & MOH-001014-00; Duke-NUS Medical School: Duke-NUS/RSF/2021/0018 & 05/FY2020/EX/15-A58; 10.13039/501100022755Agency for Science, Technology and Research: A20H4g2141 & H20C6a0032.

## Data availability statement

The data that support the findings of this study are available from the corresponding author upon reasonable request.

## Declaration of competing interest

Author DVG reports investment in DoctorBell (acquired by MaNaDr, Mobile Health), AskDr, VISRE, Healthlink, and Shyfts. He reports serving as senior lecturer and faculty advisor to the medical innovation program of NUS, and appointment as physician leader (telemedicine) and general practitioner at Raffles Medical Group (RMG, SGX:$BSL).
